# Aerobic Capacity and Restitution Efficiency Level in Relation to the Training Experience and Weekly Training Volume of Male and Female Judo National Team Members in the Cadet Age Group (U18) during the Preparatory Period

**DOI:** 10.3390/ijerph191711142

**Published:** 2022-09-05

**Authors:** Adam Prokopczyk, Marek Sokołowski

**Affiliations:** Department of Sport and Defence Education, Poznan University of Physical Education, Królowej Jadwigi Street 27/39, 61-871 Poznań, Poland

**Keywords:** capacity, preparatory period, restitution efficiency, judo, national team, cadets, martial arts, training experience, training volume

## Abstract

This study aims to analyze the relationship between the level of aerobic capacity and post-exercise restitution during the preparation period, with training experience and weekly training volume. All (12) athletes (six women; six men) participating in the training camp of the Polish national judo team in the cadet age group were tested. The Maximal Multistage 20-m Shuttle Run Test was used to investigate the level of aerobic capacity, and the Klonowicz coefficient of restitution (COR) 3 min after exercise (COR 3′) and 5 min after exercise (COR 5′) was used to determine the level of post-exercise restitution efficiency. The results showed that higher training experience significantly affected the deterioration of COR 3′ in female athletes and improved the results in the capacity test of male athletes. Female and male athletes with a higher weekly training volume had a higher HR at the end of the performance test. Considering the demonstrated correlations, special attention should be paid to matching the loads in training programs to the age of the athletes, introducing into training programs the teaching of body management during fatigue. Failing to adjust this may make participation impossible in competitions at the international level for older age groups.

## 1. Introduction

Winning at the international championship level in a sport requires from the competitors a continuous and regular improvement of their skills, and from the coaches who lead them, a regular control, and, if necessary, modification of the implemented training process. Very often, the final result of a sports competition is determined by physiological indicators. The physiological preparation cannot be left out of the training process. In order to not disturb the process of an athlete’s adaptation to loads occurring in subsequent training cycles, selected physiological indicators should be controlled [1]. In judo, regardless of the age category, such physiological indices are aerobic capacity and restitution efficiency, which often allow assessment of the athlete’s preparation in relation to the implemented training cycle. According to the Sport and Organization Rules (SOR) of the International Judo Federation (IJF), a judo fight lasts 4 min, regardless of the age group. However, if there is no winner in the regulation time of the fight, there is an overtime period called Golden Score. This overtime lasts without a time limit until the winner is determined [2]. Based on the specificity of the sport, judo is characterized by high intensity and short rests during the fight. Although the competitors obtain most of their energy from anaerobic metabolism during the fight, it is necessary to achieve a high level of aerobic capacity. Moreover, the effective shaping of the aerobic capacity is related to the use of lactic acid, which leads to better regeneration during judo fights [3,4,5], indicating the significance of aerobic capacity in the judo training process. The level of aerobic capacity in judo is used as a tool to assess cardiovascular fitness [6]. Together with COR, it should be one of the basic control tools in the training process, regardless of the age group [7]. Consequently, this parameter, together with the level of aerobic capacity, may be the basis for planning or modifying the implemented training process during the preparatory period for main competitions.

The subject of the present article concerns the influence of the training experience and weekly training volume on the level of aerobic capacity and COR among Polish Cadet (U18) team members during the preparatory period. Previous studies have analyzed factors influencing aerobic and anaerobic capacity in martial arts [8], demonstrated the influence of aerobic capacity level on the results obtained in special fitness tests in judo [5,9,10], compared aerobic capacities of judokas aged child, cadet, and senior [11], and determined the desired physiological profile of a judo athlete in the cadet age group [12]. To further analyze the level of aerobic capacity, maximal, submaximal, and resting heart rates of male and female athletes during competition [13] were analyzed in relation to their athletic performance [14]. The changes occurring in different high-intensity training modes [15] were monitored using heart rate and the recovery capacity in judokas in relation to the level of training load [16]. A specific training program for elite athletes has been developed with the aim of shaping their Vo2max levels by the additions of high-intensity interval training [17]. An analysis was performed of the differences in aerobic and anaerobic capacity levels between different age groups (senior, junior, and cadet) competing at the world level in judo. However, these studies were performed on athletes competing at the national level and during the transition period (one–two weeks after the respective main competitions) [18]. Moreover, previous research shows that the result of judo fight is significantly correlated with the level of judo special skills [19,20]. Those skills are determined by the level of aerobic capacity in the cadet age group [21]. This indicates that it is necessary to regularly analyze and shape this physiological feature at the cadet level of judo training.

The importance of this issue can be emphasized by the fact that significant correlations have been demonstrated between the training length and weekly training volume and the training efficiency and the level of restitution in female and male athletes in the preparation period for the Olympic Games [22,23]. Therefore, the authors decided to conduct an analysis of the above relationships for athletes competing at the international level in the cadet age group, which is the first age group in the arena of international competition. This study will provide an opportunity to compare athletes beginning to struggle in competitions at the level of international championships, already being in the most important period of their sports career. Moreover, as an additional element of the study (important due to the specificity of sport), the level of efficiency was related to the body mass of the subjects, which may be an important element of the training process in the age of the subjects.

In the present study, the authors posed two hypotheses:

**Hypothesis** **1 (H1).** *Athletes with more training experience will obtain better results in the aerobic capacity test*.

**Hypothesis** **2 (H2).** *Weekly training volume affects the level of effective post-exercise restitution*.

## 2. Materials and Methods

**Material.** The research was conducted among Polish Judo National Team members in the cadet group (U18) who were staying at their first preparation training camp during the preparatory period. A total of 12 competitors, 6 women and 6 men, were examined. The average age was 16.43 years for women and 16.15 years for men. A realized research limitation was the number of respondents subjected to the research. The authors, analyzing the highest-level competitors in the country, only had the opportunity to analyze a small group of players (who were in the Polish Judo National Team). During the research project, the authors did not have the possibility to conduct an analysis of competitors in the same age group and the same training period. Due to the lack of access to a similar age group, the study did not include a control group.

**Methods.** Aerobic capacity was measured using the Maximal Multistage 20-m Shuttle Run Test. During the test, the subject has to move between lines 20 m apart according to a sound signal. The test subject must cross the line before the beep, or he or she will receive a warning. If a second warning is given, the test is terminated. The test is conducted with increasing frequency of beeps [24]. During the examination, the beeps were produced by an audio device connected with the sound system in the sports hall where the examination took place. The frequency of the signals was consistent with the pace necessary for the subjects to overcome. During the conduct of the test, the distance and the level at which the subject completed the test were marked. On this basis, the level of aerobic capacity was estimated using the formula:

Vo_2_max = −32,678 + 6592 × P,

where P—the maximum speed of the distance at which the run ended (km/h) [25]. The test was carried out at the beginning of the training camp instead of the usual morning training, and on that day, the competitors had no load before. Moreover, in the morning of the same day, body weight and body height were measured (after wake-up). Previously at this camp, the competitors had one training day during which they had one low-intensity training session.

After the end of the performance test, heart rate was measured to assess the subjects’ post-exercise restitution at 3 min and 5 min. To calculate the level of restitution, Klonowicz COR was used at 3 min (COR_3′_) and 5 minutes (COR_5′_) after the exercise according to the following formulas:

$${\mathrm{COR}}_{3\prime }=\frac{Hr2-Hr3}{Hr2-Hr1}\;\mathrm{and}\;{\mathrm{COR}}_{5\prime }=\frac{Hr2-Hr5}{Hr2-Hr1},$$

where:

*Hr*_1_—resting heart rate

*Hr*_2_—heart rate measured at the end of the test

*Hr*_3_—heart rate measured at 3′ post-exercise restitution

*Hr*_5_—heart rate measured at 5′ post-exercise restitution [26].

**Ethical issues.** The authors obtained approval for the present study from the Bioethics Committee at the Poznan University of Medical Sciences, issued on 12 September 2019, number 880/19.

**Statistical methods.** Mean and standard deviation were calculated in this study. When the variables did not differ significantly from the normal distribution, Pearson’s r coefficient was used. In the case when at least one of the variables was significantly different from the normal distribution (Shapiro–Wilk W test), then the correlation coefficient R-Spearman was applied. The obtained calculation values were considered statistically significant when the value of *p* < 0.05. Moreover, due to the small size of the tested group, when the value of *p* < 0.1, the calculation value was considered borderline significant.

## 3. Results

The training experience, training volume, and values obtained in the study of female and male athletes are included in the following Table 1. It is noteworthy that in both male and female subjects, there was a significant difference in the weekly training volume and in the COR after 3 min and after 5 min, both between the extreme values and between the average and the limit values. It is important to note that there was a limit value of more than 100% of the effectiveness of restitution and the distance covered by the tested athletes during the capacity test both in the male and female groups.

Based on the W Shapiro–Wilk test, it was shown that there are variables in the study group that are significantly different from the normality distribution. In the group of women, it was Klonowicz 3′ COR, and in the group of men: Beep-Test level (by order), Beep-Test distance (m), average speed of the last level, Beep-Test (km/h), Vo_2_max by Beep-Test (ml/kg/min), and Beep-Test post-test HR (rate/min). Spearman’s r correlation was used for the above variables. For the others, Pearson’s r correlation was applied.

Using Pearson’s r correlation, there were two significant associations in the female group between weekly training volume and post-test HR (0.825; *p* = 0.043) and HR 1 min post-test (0.857; *p* = 0.029). No significant associations were shown in the male group (Table 2).

Spearman’s R correlation revealed one significant association between training seniority and Klonowicz COR 3′ in the female group (−0.829; *p* = 0.042). The men’s group showed a significant association between weekly training volume and post-test HR (0.851; *p* = 0.032) and marginal borderline significance between training experience and Beep-Test level (by order) (0.783; *p* = 0.065), mean speed of last Beep-Test level (km/h) (0.783; *p* = 0.065), and Vo_2_max by Beep-Test (ml/kg/min) (0.783; *p* = 0.065).

Considering the very small size of the study group (six athletes), the authors believe that the borderline significance presented in Table 3 should be taken into account.

## 4. Discussion

After analyzing the results, a significant effect of training seniority on COR in women and aerobic capacity level in men was shown. The effect of weekly training volume on HR level after the aerobic capacity test was also shown in both men and women. According to an analysis of judo fights at top-level tournaments, most bouts end before the end of the fight time. Athletes are increasingly imposing a fast pace that is associated with working in a different metabolic zone. However, in order to increase their chances of success, they must be able to maintain it until the end of the fight (which may not end before time and result in overtime), as well as the entire tournament [27]. This indicates that it is necessary for the athletes to have a high level of aerobic capacity and the ability to manage their effort properly in order to achieve a significant result at the main event. This can be confirmed by the results obtained in our study, in which athletes with more experience achieved significantly better results in the test of aerobic capacity, which consisted of covering the same distance at increasing intensity. These results are consistent with research indicating that younger athletes with more judo experience perform better in fitness tests and achieve better results in athletic competitions [28,29]. Importantly, in a study of elite judo athletes competing at the senior level, it was shown that training experience no longer significantly affected the results of fitness and capacity tests [30]. However, it has been indicated that the training process at the highest level should include comprehensive technical and fitness training solutions [31]. This may indicate that after gaining some training or competition experience, athletes acquire the ability to better manage their fatigue during efforts, or from a certain point in their competitive career, begin to be trained in effective body management during exertion. In the opinion of the authors, effort ergonomics should be a component of the training process, especially in the cadet age group, providing a basis for greater efficiency of training units and shaping the skills for optimizing fatigue management at senior age. Bearing in mind the small sample size of this study, the authors note that in order to better explore the issue, it would be necessary to conduct research on a larger group of subjects and analyze the number of competitions they have participated in, the training methods and forms they have experienced, and the diversity of the environments in which they trained and participated in competitions (which may affect adaptive changes of the body). Higher weekly training loads were correlated with an increase of HR at the end of the test for both male and female athletes. Furthermore, in female athletes, weekly training volume had a significant effect on increasing HR 1 min after the end of the capacity test. As the weekly training volume is similar to that of the Polish National Team [22,23] and did not show any significance indicating an increase in the subjects’ performance level, the authors believe that the subjects are either overtrained or their training process is inadequately adapted to their age. Previous research indicates that judo players in the cadet age group should pursue a training process focused on motricity, technical-tactical, and mental development, as they are the determinants in fights at the cadet level [32,33]. Specialization in this age group and the implementation of training programs from the senior level, despite the achievement of sports results, significantly limits the possibility of succeeding in senior age [34]. Moreover, based on biochemical research and HR analysis, it has been proven that 15-min breaks between consecutive fights during judo competitions in the cadet age group should be adequate for regeneration between them. During this time, the athlete should regenerate physiologically and metabolically [35]. Bearing in mind the correlation with the weekly training volume and the significant correlation of the training duration decreasing the level of post-exercise COR in female athletes, it can be assumed that female athletes who train in judo longer are subjected to training programs dedicated to older athletes. In order to achieve the assumed training goals, continuous observation of proper training periodization is necessary. To help athletes develop, it is necessary to adjust training to their age and individual predispositions during puberty. Older elite athletes of both sexes have a better physiological predisposition to endure higher level and volume loads [36] and they have a higher resilience, which often determines an athlete’s training capabilities [37]. As previous studies have shown, the balance between training load and recovery significantly influences the effects of implemented training programs both in cadet-age judo athletes and in elite seniors [38,39]. Consequently, the training process for the cadet age group must be adjusted to the training period in the long-term plan, the level of individual motor abilities, and the experience of the athlete. Moreover, it is very important to conduct mental preparation within the training process in the cadet age group, which often determines the effect of undertaken efforts, judo fights, as well as the regeneration process [40,41].

The studies did not show a significant correlation between relative Vo2max index and body weight. In the opinion of the authors, this may be due to the small study group and no analysis of their body composition, as many studies have shown the Vo_2_max level is related to the body composition of fighters training in martial arts. Previous studies indicate the relationship between fat mass, free fat mass and Vo_2_max in both groups of adolescent and adult competitors [42]. The highest Vo_2_max was recorded in competitors with the lowest fat mass, and a decrease in Vo_2_max was observed with an increase in fat mass [43,44,45]. In addition, studies analyzing judo players at the champion level indicate that they are characterized by an increase in body weight related of muscular hypertrophy and a decrease of fat mass [46]. Considering the above, the authors believe that further studies of this type should be conducted with the analysis of body composition. It will give the possibility a comprehensive assessment of the aerobic capacity of players.

## 5. Conclusions

Research indicates that greater training seniority improves performance in the capacity test in males, although it does not significantly affect the results obtained by women. The research shown that higher weekly training volume worsens COR in women and does not significantly affect post-exercise restitution in men. Considering that higher HR scores after the aerobic test were influenced by higher weekly training volumes (women and men) and the deterioration of effective post-exercise restitution was influenced by greater competition seniority (women), it can be assumed that the studied athletes were in a state of overtraining and premature specialization in judo. Taking into account the limitations of this study, the authors indicate the need for future research incorporating the analysis of body composition and involving a large group of respondents.

## Figures and Tables

**Table 1:** Body weight, body height, training experience, weekly training volume, Beep-Test scores, and COR of female and male judo cadet national team athletes (U18).

Variable	Average	Median	Minimum	Maximum	Standard Deviation
**Female**					
Body weight (kg)	60.5	61.6	44.2	77	12.9
Body height (cm)	166.3	170	153	180	11
Training experience (years)	9.2	9.5	7	11	1.6
Weekly training volume (h)	15.92	16	7.5	22	5.1
Level in Beep-Test (in order of level)	8.2	8	7	9	0.8
Distance in Beep-Test (m)	1363.3	1310	1140	1620	188.2
Average running speed of the last level of Beep-Test (km/h)	12.1	12	11.5	12.5	0.3
Vo_2_max based on Beep-Test (mL/kg/min)	47.0	46.4	43.1	49.7	2.5
Resting HR (bpm)	72.7	73	58	90	12.4
Beep-Test—HR after the test (bpm)	150.3	158	120	172	23.6
Beep-Test—HR 1′ after the test (bpm)	123	120	100	156	22.5
Beep-Test—HR 3′ after the test (bpm)	106.3	102	80	134	18.6
Beep-Test—HR 5′ after the test (bpm)	91.3	89	64	126	23.6
Klonowicz COR 3′	52.3	63.3	11.8	68	22.2
Klonowicz COR 5′	74.1	77.3	41.2	105.4	24.7
Relative distance of Beep-Test (m/body weight)	23.6	24	24.0	16.1	32.5
Relative average running speed of the last level of Beep-Test [(m/h/body weight)	0.2	0.2	0.2	0.2	0.3
Relative Vo_2_max based on Beep-Test (mL/kg/min/body weight)	0.8	0.8	0.8	0.6	1.1
**Male**					
Body weight (kg)	78.9	76.5	57.5	110	18.9
Body height (cm)	178.5	182.5	161	188	10.8
Training experience (years)	9.8	10	9	11	0.8
Weekly training volume (h)	11.3	11.5	7	14.5	2.4
Level in Beep-Test (in order of level)	9.8	10.5	6	11	1.9
Distance in Beep-Test (m)	1723.3	1830	980	2020	1740
Average running speed of the last level of Beep-Test (km/h)	12.9	13.3	11	13.5	0.9
Vo_2_max based on Beep-Test (mL/kg/min)	52.5	54.7	39.8	56.3	6.4
Resting HR (bpm)	84.7	83	74	102	10.4
Beep-Test—HR after the test (bpm)	159	165	128	168	15.5
Beep-Test—HR 1′ after the test (bpm)	133.7	132	120	150	14.0
Beep-Test—HR 3′ after the test (bpm)	115	116	106	124	6.3
Beep-Test—HR 5′ after the test (bpm)	95.7	98	76	112	16.2
Klonowicza COR 3′	57.9	59.5	22.2	78.9	20.5
Klonowicza COR 5′	85.4	81	65.2	118.4	21.0
Relative distance of Beep-Test (m/body weight)	23.6	24.1	24.1	8.9	33.7
Relative average running speed of the last level of Beep-Test (km/h/body weight)	0.2	0.17	0.2	0.1	0.2
Relative Vo_2_max based on Beep-Test (mL/kg/min/body weight)	0.7	0.7	0.7	0.4	1.0

**Table 2:** Training experience and weekly training volumes versus Beep-Test scores and COR of female and male judo cadet (U18) national team athletes using Pearson’s r correlation (for variables not significantly deviating from the normal distribution).

Variable	Training Experience	Weekly Training Volume
**Female**		
Level in Beep-Test (in order of level)	−0.599; *p* = 0.209	0.319; *p* = 0.537
Distance in Beep-Test (m)	−0.577; *p* = 0.230	0.347; *p* = 0.501
Average running speed of the last level of Beep-Test (km/h)	−0.599; *p* = 0.209	0.319; *p* = 0.537
Vo_2_max based on Beep-Test (mL/kg/min)	−0.599; *p* = 0.209	0.319; *p* = 0.537
Resting HR (bpm)	0.349; *p* = 0.497	−0.283; *p* = 0.587
Beep-Test—HR after the test (bpm)	0.169; *p* = 0.749	0.825; *p* = 0.043
Beep-Test—HR 1′ after the test (bpm)	0.170; *p* = 0.748	0.857; *p* = 0.029
Beep-Test—HR 3′ after the test (bpm)	0.677; *p* = 0.140	0.070; *p* = 0.895
Beep-Test—HR 5′ after the test (bpm)	0.619; *p* = 0.190	0.065; *p* = 0.903
Klonowicza COR 5′	−0.605; *p* = 0.203	0.289; *p* = 0.579
Relative distance of Beep-Test (m/body weight)	0.147; *p* = 0.782	0.274; *p* = 0.599
Relative average running speed of the last level of Beep-Test (km/h/body weight)	0.441; *p* = 0.382	0.205; *p* = 0.696
Relative Vo_2_max based on Beep-Test (mL/kg/min/body weight)	0.374; *p* = 0.466	0.222; *p* = 0.673
**Male**		
Resting HR (bpm)	−0.136; *p* = 0.797	0.598; *p* = 0.210
Beep-Test—HR 1′ after the test (bpm)	0.411; *p* = 0.418	−0.389; *p* = 0.446
Beep-Test—HR 3′ after the test (bpm)	0.211; *p* = 0.688	0.293; *p* = 0.574
Beep-Test—HR 5′ after the test (bpm)	0.158; *p* = 0.764	0.532; *p* = 0.277
Klonowicz COR 3′	0.145; *p* = 0.784	0.255; *p* = 0.626
Klonowicz COR 5′	−0.126; *p* = 0.813	−0.144; *p* = 0.786
Relative distance of Beep-Test (m/body weight)	0.638; *p* = 0.173	−0.225; *p* = 0.668
Relative average running speed of the last level of Beep-Test (km/h/body weight)	0.629; *p* = 0.181	−0.180; *p* = 0.733
Relative Vo_2_max based on Beep-Test (mL/kg/min/body weight)	0.640; *p* = 0.551	−0.206; *p* = 0.695

**Table 3:** Training experience and weekly training volume versus Beep-Test scores and COR of female and male judo cadet (U18) national team athletes using Spearman’s r correlation (only for variables deviating significantly from the normal distribution).

Variable	Training Experience	Weekly Training Volume
**Female**		
Klonowicz COR 3′	−0.829; *p* = 0.042	0.464; *p* = 0.354
**Male**		
Level in Beep-Test (in order of level)	0.783; *p* = 0.065	−0.080; *p* = 0.881
Distance in Beep-Test (m)	0.610; *p* = 0.198	0.045; *p* = 0.933
Average running speed of the last level of Beep-Test (km/h)	0.783; *p* = 0.065	−0.080; *p* = 0.881
Vo_2_max based on Beep-Test (mL/kg/min)	0.783; *p* = 0.065	−0.080; *p* = 0.881
Beep-Test—HR after the test (bpm)	0.018; *p* = 0.977	0.851; *p* = 0.032

## Data Availability

Data available on request due to confidentiality of the results of specific competitors of the Polish Judo National Team.
